# Red blood cell distribution width (RDW) is a significant predictor of survival in laryngeal cancer patients: Systematic literature review and meta-analysis

**DOI:** 10.5937/jomb0-42947

**Published:** 2023-10-27

**Authors:** Riccardo Nocini, Fabian Sanchis-Gomar, Giuseppe Lippi, Camilla Mattiuzzi

**Affiliations:** 1 University of Verona, Department of Surgery, Dentistry, Pediatrics and Gynecology-Unit of Otorhinolaryngology, Verona, Italy; 2 Stanford University School of Medicine, Division of Cardiovascular Medicine, Stanford, United States; 3 University of Verona, Section of Clinical Biochemistry and School of Medicine, Verona, Italy; 4 Provincial Agency for Social and Sanitary Services (APSS), Service of Clinical Governance and Medical Direction, Trento, Italy

**Keywords:** RDW, red blood cell distribution width, anisocytosis, laryngeal cancer, larynx cancer, RDW, širina distribucije crvenih krvnih zrnaca, anizocitoza, rak larinksa, rak larinksa

## Abstract

**Background:**

This systematic literature review and meta-analysis investigated whether the red blood cell distribution (RDW) may predict survival outcomes in laryngeal cancer patients undergoing curative treatment.

**Methods:**

We conducted an electronic search in Medline and Scopus using the keywords "red blood cell distribution width" OR "RDW" AND "laryngeal cancer" OR "larynx cancer" OR "laryngeal carcinoma" OR "larynx carcinoma," without time or language restrictions (up to February 2023), for identifying studies investigating the prognostic value of RDW in patients with any form of laryngeal cancer and with a primary endpoint that was set as survival rate and/or disease-free survival between 1 and 10 years after curative treatment. The research was conducted according to the PRISMA (Preferred Reporting Items for Systematic Reviews and Meta-Analyses) 2020 reporting checklist.

## Abstract (sr)

**Metode:**

Izvršili smo elektronsku pretragu u Medline i Scopusu koriste}i ključne reći "širina distribucije crvenih krvnih zrnaca" ILI "RDW" I "rak larinksa" ILI "karcinom larinksa" ILI "karcinom larinksa" ILI "karcinom larinksa", bez vremenskih ili jezičkih ograničenja (do februara 2022. godine), za identifikaciju prognostičkih vrednosti RDW u pacijenata sa bilo kojim oblikom laringealnog kancera i sa primarnom krajnjom tačkom koja je postavljena kao stopa preživljavanja i/ili preživljavanje bez bolesti izme|u 1 i 10 godina nakon kurativnog lečenja. Istraživanje je sprovedeno prema PRISMA (Preferred Reporting Items for Sistematic Revievs and Meta-Analises) kontrolnoj listi za izveštavanje za 2020. godinu.

**Rezultati:**

Digitalna pretraga nam je omogućila da identifikujemo pet studija koje su konačno uključene u na{u objedinjenu analizu (stopa preživljavanja i/ili preživljavanje bez bolesti pokrivali su period izmđu 2,7-8,3 godine). U četiri od pet studija, povećana vrednost RDW kod pacijenata sa karcinomom larinksa koji su bili podvrgnuti hirurškom tretmanu ili tretmanu zračenjem bila je povezana sa lošijim preživljavanjem (opseg odnosa šanse ORs, 2,28-10,44). U objedinjenoj analizi, povećani RDW je dao preko 3 puta veći rizik od umiranja tokom praćenja nakon kurativnog lečenja karcinoma larinksa (OR, 3,37; 95% CI, 1,41-8,10).

**Zaključak:**

RDW zadržava klinički važnu prognostičku vrednost kod pacijenata sa karcinomom larinksa koji su podvrgnuti kurativnom lečenju. Kliničari bi mogli da priznaju ove informacije za korišćenje agresivnije terapije ili postavljanje užeg praćenja kod pacijenata sa rakom larinksa sa povećanim vrednostima RDW.

## Introduction

Laryngeal cancer, a malignant neoplasm originating from the larynx tissues, represents nearly onethird of all head and neck cancers and is now regarded as an important public health issue. Besides being listed among the 20 most deadly cancer worldwide [1], the prevalence of laryngeal cancer is around 14 cases per 100,000, following a dramatically increase (by 24%) over the past 3 decades [2], with a relative contextual surge of 25% in the case-fatality rate [3].

Larynx cancer detected at an early stage can be successfully treated with curative surgery, enabling local control rates between 80–95%, while regionally-advanced larynx cancers are associated with a much lower control rate, between 40–70% [4]. Although the survival rate may depend on cancer location (i.e., supraglottis, glottis, or subglottis) and staging, the survival rate is as high as 80% in patients with localized laryngeal cancers, but decreases to 46% in those with tumor spread to nearby tissues and/or the regional lymph nodes, and is as low as 34% in those with metastatic cancer at the time of the diagnosis [5]. Unlike other types of cancer where important clinical and laboratory predictors have been identified and validated [6], recent evidence suggests that additional efforts are needed to identify reliable and accurate biomarkers which may help predicting the risk of unfavorable progression of patients with larynx cancer, feeble treatment response and higher likelihood of death [7].

The red blood cell distribution width (RDW) is a simple hematologic index that reflects anisocytosis and can be calculated (in percent value) from the mean and standard deviation of the erythrocyte volumes (mean corpuscular volume; MCV) using the common formula: (SD of MCV)/(MCV)×100. All modern hematological analyzers automatically generate this measure within the conventional whole blood count. It thus comes with high speed, high throughput, and no additional costs [8]. Several lines of evidence now attest that a basal RDW value, as well as its changes over time, may be important predictors of outcomes in patients with a kaleidoscope of clinical conditions, including cardiovascular disorders, diabetes, liver and kidney failure, infections, as well as in those with cancer [8] [9]. In particular, a comprehensive meta-analysis recently published by Hu et al. [10] revealed that, in general patients with different forms of malignant tumors, an augmented RDW was associated with cumulatively poor survival (hazard ratio (HR), 1.47; 95%CI, 1.29–1.66), reduced cancer-specific survival (HR, 1.46; 95%CI, 1.08–1.85), lower disease-free survival (HR, 1.91, 95%CI, 1.27–2.56), inferior event-free survival (HR, 2.98; 95%CI, 0.57–5.39) as well as poor progress-free survival (HR, 3.21; 95%CI, 0.33–6.75) after treatment. In a subsequent meta-analysis published by Wang et al. [11] in 2019, the overall survival of patients with different types of cancer was reduced by nearly 50% (HR, 1.51; 95%CI, 1.39–1.64) when the RDW value was above a certain defined threshold. Importantly, both meta-analyses did not include laryngeal cancer among the various malignancies, whilst no other systematic literature reviews have been published for specifically analyzing the significance of measuring RDW for assessing the prognosis of laryngeal cancer patients to the best of our knowledge. Therefore, we aim to assess whether RDW values may significantly predict survival outcomes in laryngeal cancer patients using a systematic literature review and meta-analysis.

## Materials and methods

We conducted an electronic search in Medline (PubMed interface) and Scopus, using the keywords »red blood cell distribution width« OR »RDW« AND »laryngeal cancer« OR »larynx cancer« OR »laryngeal carcinoma« OR »larynx carcinoma« in the fields (Article Title) OR (Abstract) OR (Keywords), without using time or language restrictions (i.e., up to February 2023), aimed at identifying all clinical studies which investigated the prognostic value of RDW in patients with any form of laryngeal cancer and with a primary endpoint that was set as survival rate and/or disease-free survival between 1 and 10 years after curative treatments. Two authors (G.L. and C.M.) screened all originally retrieved items by title, abstract, and full text (if available), selecting all studies where full data on patient survival (or death) could be retrieved from the text, figures (e.g., Kaplan-Mayer curves) or from the supplementary material, thus allowing to construct a 2×2 table where survival (Yes/No) rate could be dichotomized according to »low« or »high« RDW values. The reference list of all articles was also hand-search to identify additional suitable material. All selected studies and their leading findings were narratively described in the text. Moreover, when sufficient information was available, data were pooled to calculate the odds ratio (OR) and its 95% confidence interval of the risk of poor postoperative survival stratifying according to (dichotomous) RDW values. The heterogeneity among the selected studies was assayed with c2 test and I2 statistic, whilst a funnel plot was constructed to evaluate publication bias.

**Figure 1:**
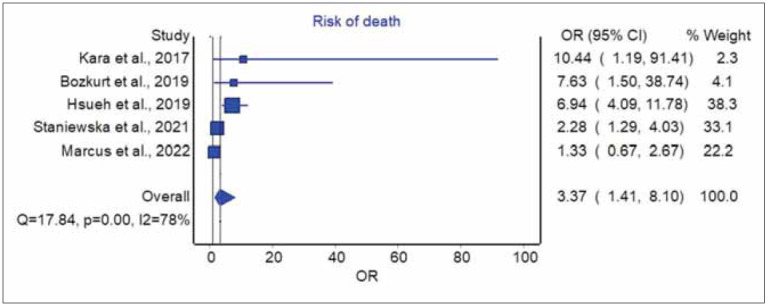
Pooled analysis of studies exploring the predictive role of red blood cell distribution width (RDW) in laryngeal cancer patients undergoing curative therapy. Results are shown as odds ratio (OR) and 95% confidence interval (95% CI).

**Figure 2:**
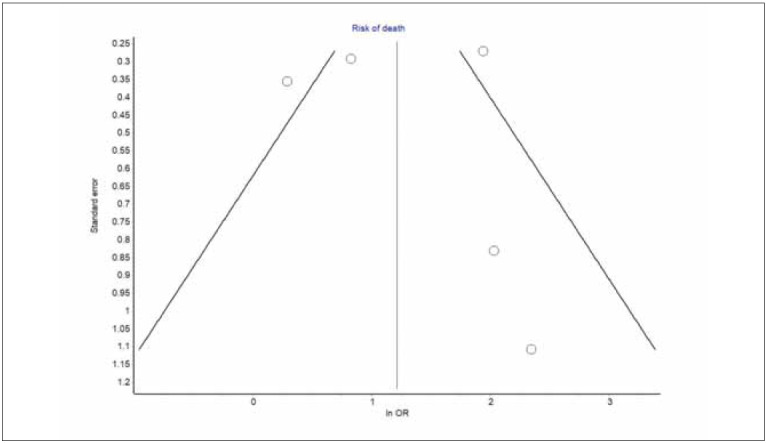
Funnel plot of studies exploring the predictive role of red blood cell distribution width (RDW) in laryngeal cancer patients undergoing curative therapy. Results are shown as odds ratio (OR).

The statistical analysis was conducted using MetaXL, Version 5.3 (EpiGear International Pty Ltd., Sunrise Beach, Australia), by calculation of the odds ratio (OR) with its 95% confidence interval (95%CI). This analysis was conducted in accordance with the declaration of Helsinki, within the term of local legislation, according to the PRISMA (Preferred Reporting Items for Systematic Reviews and Meta-Analyses) 2020 reporting checklist (Table 1). Figure 1 Figure 2

**Table 1:** Preferred Reporting Items for Systematic Reviews and Meta-Analyses (PRISMA) Checklist. From: Page MJ, McKenzie JE, Bossuyt PM, Boutron I, Hoffmann TC, Mulrow CD, et al. The PRISMA 2020 statement: an updated guideline for reporting systematic reviews. BMJ 2021;372:n71. doi: 10.1136/bmj.n71<br>For more information, visit: http://www.prisma-statement.org/

Section and Topic C	Item<br>#	Checklist item	Location where<br>item is reported
TITLE			
Title	1	Identify the report as a systematic review.	Page 1
ABSTRACT			
Abstract	2	See the PRISMA 2020 for Abstracts checklist.	Page 2
INTRODUCTION			
Rationale	3	Describe the rationale for the review in the context of existing knowledge.	Page 4–5
Objectives	4	Provide an explicit statement of the objective(s) or question(s) the review addresses.	Page 5
METHODS			
Eligibility criteria	5	Specify the inclusion and exclusion criteria for the review and how studies were<br>grouped for the syntheses.	Page 5–6
Information sources	6	Specify all databases, registers, websites, organizations, reference lists, and other sources<br>searched or consulted to identify studies. Specify the date when each source was last<br>searched or consulted.	Page 5–6
Search strategy	7	Present the full search strategies for all databases, registers, and websites, including<br>any filters and limits used.	Page 5–6
Selection process	8	Specify the methods used to decide whether a study met the inclusion criteria of the <br>review, including how many reviewers screened each record and each report retrieved, <br>whether they worked independently, and if applicable, details of <br>automation tools used in the process.	Page 5–6
Data collection<br>process	9	Specify the methods used to collect data from reports, including how many reviewers <br>collected data from each report, whether they worked independently, any processes for <br>obtaining or confirming data from study investigators, and if applicable, details of <br>automation tools used in the process.	Page 5–6
Data items	10a	List and define all outcomes for which data were sought. Specify whether all results that<br>were compatible with each outcome domain in each study were sought (e.g. for all meas-<br>ures,time points, analyses), and if not, the methods used to decide which results to collect.	Page 6
	10b	List and define all other variables for which data were sought (e.g. participant and<br>intervention characteristics, funding sources). Describe any assumptions made about<br>any missing or unclear information.	Page 6
Study risk of bias<br>assessment	11	Specify the methods used to assess risk of bias in the included studies, including details<br>of the tool(s) used, how many reviewers assessed each study and whether they worked<br>independently, and if applicable, details of automation tools used in the process.	Page 6
Effect measures	12	Specify for each outcome the effect measure(s) (e.g. risk ratio, mean difference)<br>đused in the synthesis or presentation of results.	Page 6
Synthesis methods	13a	Describe the processes used to decide which studies were eligible for each synthesis (e.g.<br>tabulating the study intervention characteristics and comparing against the planned groups<br>for each synthesis (item #5)).	Page 6
	13b	Describe any methods required to prepare the data for presentation or synthesis, such as<br>handling missing summary statistics, or data conversions.	Page 6
	13c	Describe any methods used to tabulate or visually display results of individual studies and<br>syntheses.	Page 6
	13d	Describe any methods used to synthesize results and provide a rationale for the choice(s).<br>If meta-analysis was performed, describe the model(s), method(s) to identify the presence<br>and extent of statistical heterogeneity, and software package(s) used.	Page 6
	13e	Describe any methods used to explore possible causes of heterogeneity among study results<br>(e.g. subgroup analysis, meta-regression).	Page 6
	13f	Describe any sensitivity analyses conducted to assess robustness of the synthesized results.	N/A
Reporting bias<br>assessment	14	Describe any methods used to assess risk of bias due to missing results in a synthesis<br>(arising from reporting biases).	Page 6
Certainty assessment	15	Describe any methods used to assess certainty (or confidence) in the body of evidence<br>for an outcome.	Page 6
RESULTS			
Study selection	16a	Describe the results of the search and selection process, from the number of records identified<br>in the search to the number of studies included in the review, ideally using a flow diagram.	Page 7;<br>Table 1
	16b	Cite studies that might appear to meet the inclusion criteria, but which were excluded,<br>and explain why they were excluded.	Page 7–9
Study characteristics	17	Cite each included study and present its characteristics.	Page 7–8
Risk of bias in studies	18	Present assessments of risk of bias for each included study.	Figure 2
Results of individual<br>studies	19	For all outcomes, present, for each study: (a) summary statistics for each group (whereappropriate) and (b) an effect estimate and its precision (e.g. confidence/credible interval),ideally using structured tables or plots.	Figure 1;<br>Page 10
Results of syntheses	20a	For each synthesis, briefly summarise the characteristics and risk of bias among<br>contributing studies.	Page 7-8
	20b	Present results of all statistical syntheses conducted. If meta-analysis was done, present for<br>each the summary estimate and its precision (e.g. confidence/credible interval) and measures<br>of statistical heterogeneity. If comparing groups, describe the direction of the effect.	Page 7–10;<br>Figure 1
	20c	Present results of all investigations of possible causes of heterogeneity among study results.	Page 8
	20d	Present results of all sensitivity analyses conducted to assess the robustness of the<br>synthesized results.	N/A
Reporting biases	21	Present assessments of risk of bias due to missing results (arising from reporting biases) for<br>each synthesis assessed.	Figure 2
Certainty of evidence	22	Present assessments of certainty (or confidence) in the body of evidence for each outcome<br>assessed.	N/A
DISCUSSION			
	23a	Provide a general interpretation of the results in the context of other evidence.	Page 10–12
	23b	Discuss any limitations of the evidence included in the review.	Page 12
	23c	Discuss any limitations of the review processes used.	Page 12
	23d	Discuss implications of the results for practice, policy, and future research.	Page 11–12
OTHER INFORMATION			
Registration and<br>protocol	24a	Provide registration information for the review, including register name and<br>registration number, or state that the review was not registered.	N/A
	24b	Indicate where the review protocol can be accessed, or state that a protocol<br>was not prepared.	N/A
	24c	Describe and explain any amendments to information provided at registration<br>or in the protocol.	N/A
Support	25	Describe sources of financial or non-financial support for the review, and the role of the fun-<br>dersor sponsors in the review.	Page 12
Competing interests	26	Declare any competing interests of review authors.	Page 12
Availability of data,<br>code and other<br>materials	27	Report which of the following are publicly available and where they can be found:<br>template data collection forms; data extracted from included studies; data used for all analy-<br>ses;analytic code; any other materials used in the review.	Upon request to corr. author

## Results

### Study selection

The digital search based on the criteria mentioned above enabled the identification of 12 articles after eliminating redundancy between the two scientific repositories. Of these, 4 articles ought to be excluded because they did not show sufficient information on RDW values for being included in the pooled analysis, and 3 because they failed to report RDW data in patients with laryngeal cancer. Thus, five studies could be finally included in our pooled analysis (Table 2) [12] [13] [14] [15] [16]. All these studies were retrospective in nature; 2 were conducted in Turkey and one each in China, Poland, and USA; 4 studies included laryngeal cancer patients undergoing curative surgery and one undergoing radiotherapy; the cutoff of RDW used in the different studies was comprised between 13.5–14.5%. All studies used survival rate and/or disease-free survival as primary outcomes over a time window ranging between 2.7–8.3 years (Table 2).

**Table 2:** Summary of all studies exploring the predictive role of red blood cell distribution width (RDW) in laryngeal cancer patients undergoing curative therapy. RDW, red blood cell distribution width.

Authors	Setting	Study design	Population	Outcome used for<br>the pooled analysis	Findings
Kara et al.,<br>2017 [12]	Turkey	Retrospective<br>study	Laryngeal cancer patients<br>(n=81) undergoing<br>curative surgery	Cumulative<br>survival at<br>2.7±3.3 years	Cumulative survival lower in those<br>with RDW values <14.05% compared<br>to those with higher values
Bozkurt et al.,<br>2019 [13]	Turkey	Retrospective<br>study	Laryngeal cancer patients<br>(n=132) undergoing<br>curative surgery	Disease-free<br>survival at<br>8.3 years	Disease free survival lower in those<br>in the highest (>14.4%) compared<br>to those in the lowest (<13%)<br>RDW quartile
Hsueh et al.,<br>2019 [14]	China	Retrospective<br>study	Laryngeal squamous cell<br>carcinoma patients<br>(n=809) undergoing<br>curative surgery	Cumulative<br>survival<br>at 5 years	Cumulative survival lower<br>in those in the highest<br>(>13.5%) RDW quartile
Staniewska et<br>al., 2021 [15]	Poland	Retrospective<br>study	Oropharyngeal cancer<br>patients (n=208) treated<br>with radiotherapy	Cumulative<br>survival after<br>5 years	Cumulative survival was lower in<br>those with RDW >13.8% compared<br>to those with lower values
Marcus et al.,<br>2022 [16]	USA	Retrospective<br>study	Laryngeal squamous<br>cell carcinoma patients<br>(n=177) undergoing<br>curative surgery	Cumulative<br>survival<br>at 5 years	Overall survival similar in those with<br>RDW values <14.5% or 14.5%

### Narrative description

In 2017 Kara et al. [12] retrospectively studied 81 laryngeal cancer patients undergoing curative laryngectomy, who were followed up for a mean period of 2.7±3.3 years. The cumulative survival was significantly lower in patients with RDW values <14.05% compared to those with higher values (81.8% vs. 97.9%; p=0.012). Accordingly, patients who survived after curative surgery displayed lower RDW values than those who died (i.e., RDW 14.00±1.21 vs. 14.84±0.87 %; p=0.031).

Bozkurt et al. [13] in 2019 conducted a retrospective study including 132 laryngeal cancer patients undergoing curative surgery. The primary outcome (i.e., disease-free survival at 8.3 years) was found to be lower in patients in the highest (>14.4%) compared to those in the lowest (<13.0%) RDW quartile. Moreover, an RDW value >14.4% at diagnosis significantly predicted higher risk of locoregional cancer recurrence (HR, 5.82; 95%CI, 1.25–26.97).

In the same year, Hsueh et al. [14] published a retrospective study including 809 laryngeal squamous cell carcinoma patients undergoing curative surgery. The cumulative survival at 5 years of such patients was significantly lower in those in the highest (>13.5%) RDW quartile. Specifically, patients with RDW >13.5% had a 67% higher risk of dying (HR, 1.67; 1.44–1.94) than those with RDW ≤12.9%. Notably, an identical association was found with disease-free survival (HR, 1.78; 95%CI, 1.52–2.07) and cancer-specific survival (HR, 1.74; 1.48–2.06).

Staniewska et al. [15] concluded a retrospective study including 208 oropharyngeal cancer patients undergoing radiotherapy and followed up for a median of 3.8 years. In univariate analysis, the cumulative survival was significantly reduced in those with RDW >13.8% compared to those with lower values (HR, 1.28; 95%CI, 1.12–1.47). Overall, the RDW displayed a good accuracy for predicting unfavorable outcome (area under the curve (AUC), 0.59; 95%CI: 0.51–0.67; p=0.02).

In 2022, Marcus et al. [16] reported the results of another retrospective study, including 177 patients with laryngeal squamous cell carcinoma patients undergoing laryngectomy who were followed up for 15 years. The medium- and long-term survival rate at 5 years did not differ significantly between patients with RDW values <14.5% or ≥14.5% (p=0.415 and p=0.950, respectively). Nonetheless, the authors found an over 4-fold higher prevalence (29% vs. 7%; p<0.001) of postoperative systematic complications (i.e., venous thrombosis and other cardiovascular events, pneumonia, dependence on mechanical ventilation) in patients with higher RDW values.

Some of the studies we omitted from our pooled analysis because they did not fulfill our preset inclusion criteria deserve mentioning. On the assumption that both high RDW values and low body mass index (BMI) were found to be predictive of unfavorable outcomes in patients with several types of cancer, Fu et al. [17] developed a prognostic score called COR-BMI (Combination of Red Blood Cell Distribution Width and Body Mass Index), which was tested in a retrospective study including 807 laryngeal squamous cell carcinoma patients undergoing curative surgery. The cancer-specific survival rate was found to be substantially lower in those with a COR-BMI score of 2 (i.e., RDW >13.1 and BMI <18.5) compared to those with a COR-BMI score of 0 (RDW ≤13.1 and BMI ≥25) throughout all the time points (5-year: 57% vs. 81%; 10-year: 40% vs. 79%; 15-year: 40% vs. 79%, respectively). Overall, a COR-BMI score of 2 was associated with a nearly 3-fold lower likelihood of being alive throughout the study period than a COR-BMI score of 0 (HR, 2.91; 95%CI, 1.53–5.54). The study published by Shen et al. [18], which was also omitted from our analysis since it failed to present sufficient information to construct a 2×2 table of dead and survived laryngeal cancer patients, retrospectively analyzed the data of 338 patients with laryngeal carcinoma undergoing curative surgery. Cumu latively, the RDW value (patients were stratified as having RDW ≤12.9% or >12.9%) was not found to be a significant predictor of overall survival (HR, 1.01; 95%CI, 0.72–1.41; p=0.961).

### Pooled analysis

The pooled analysis of individual data presented in the five studies that could be included in this article is shown in Figure 1. In four of the five studies included in our analysis, an enhanced RDW value in laryngeal cancer patients undergoing surgical or radiation treatment was associated with poorer survival (range of ORs, 2.28–10.44). In the pooled analysis, increased RDW conferred an over 3-fold higher risk of dying during follow-up after surgical or radiation treatment for laryngeal cancer (OR, 3.37; 95%CI, 1.41–8.10). These results remained almost unvaried after eliminating the only study where radiotherapy was used as primary curative treatment (OR, 4.09; 95%CI, 1.11–15.13). High heterogeneity was found among studies (I2, 78%; 95%CI, 46–91%), with a relatively low publication bias (Figure 2).

## Discussion

The use of RDW has now become commonplace across many clinical settings since this simple, fast, and straightforward surrogate measure of erythrocyte anisocytosis possesses a considerably high discriminatory and predictive value in the complex and multifaceted prognostication process of many human pathologies [19]. The fact that the RDW value may be an important predictor of cancer prognosis is not new since this association has been previously demonstrated in patients with different types of malignancy [10] [11]. Taken together, the results of our analysis suggest that RDW may also be used for garnering useful information on the survival rate and/or disease-free survival of patients with laryngeal cancers undergoing curative treatment. To this end, we found that larynx cancer patients with enhanced RDW values have an over 3-fold higher likelihood of medium-term mortality (i.e., within 10 years) than those with normal/lower values. Notably, although such association was not found in the study of Shen et al. [18], it is noteworthy that the authors used an extremely low cutoff (i.e., 12.9%, selected for being the median value of their population), which is comprised within the normal reference range of most clinical laboratories and, therefore, is seemingly too low for accurately stratifying the postoperative risk. Thus, it is conceivable that using a higher RDW threshold value, as in all the other studies (i.e., 13.5% or higher), would have enabled to more accurately stratifying the patients. Even in the study by Marcus et al. [16], an enhanced RDW was not associated with decreased survival after laryngectomy. However, it was more commonly seen in patients with systemic complications after laryngectomy. As clearly stated by the authors, the time of RDW measurement differed in relation to the surgical treatment date, which may have thus contributed to bias in the final observations.

Some plausible biological justifications may be supportive of our findings. Irrespective of the fact that it remains to be finally elucidated whether anisocytosis is an active player or a simple bystander in many human pathologies, an increased variation of erythrocytes volumes (as reflected by higher RDW values) is often the expression of impaired or even disrupted erythropoiesis [20]. Erythropoiesis impairments are frequently found in cancer patients due to the convergence of many altered biological pathways, namely peritumoral inflammation, oxidative stress, poor nutritional status, and/or cachexia (leading to folate and vitamin B deficiencies), along with bone marrow neoplastic colonization. All these factors would contribute to the generation of abnormal red blood cells, characterized by heterogeneous volumes and abnormal functions (namely impaired oxygen transportation and reduced oxygenation of peripheral tissues and organs), thus finally representing a hallmark of patients with less favorable prognosis and, contextually, carriers of poor or inefficient tissue oxygenation, thus increasing the risk of these patients to progress towards an unfavorable outcome [8].

In conclusion, although the objective retrospective nature of all the five studies included in our meta-analysis does not enable us to make definitive conclusions and prospective trials would be needed to corroborate our findings, our results are seemingly showing that RDW may retain a clinically crucial prognostic value in patients with laryngeal cancer undergoing curative treatment. This information could be acknowledged by clinicians in charge of treating these patients, prompting a more aggressive therapy or setting narrower follow-up in those with increased RDW.

## Dodatak

### Research funding

None declared.

### Author contributions

All authors have accepted responsibility for the entire content of this manuscript and approved its submission.

### Informed consent

Not pertinent.

### Acknowledgments

None.

### Conflict of interest statement

All the authors declare that they have no conflict of interest in this work.
